# Short-Term Acceptability of the Woman's Condom among Married Couples in Shanghai

**DOI:** 10.1155/2016/6201421

**Published:** 2016-07-28

**Authors:** Junqing Wu, Zirong Huang, Patricia S. Coffey, Maggie Kilbourne-Brook

**Affiliations:** ^1^Key Laboratory of Reproduction Regulation of NPFPC, SIPPR, IRD, Fudan University, Shanghai, China; ^2^Obstetrics and Gynaecology Hospital, The Fudan University Medical Centre, Shanghai, China; ^3^PATH, Seattle, WA 98121, USA

## Abstract

*Background*. The Woman's Condom, a second-generation female condom designed for acceptability, is poised for introduction in China.* Method*. This single-arm study was conducted among 60 couples in China in 2010 to assess acceptability of the Woman's Condom.* Results*. Male participants reported that ease of handling, inserting, and removing the device improved significantly from first to fourth use. Female and male participants reported that comfort during insertion, feel of lubricant during insertion, comfort/fit of outer ring during use, and overall comfort improved significantly from first to fourth use. Further, at fourth use, female participants reported significant improvement in the comfort of the feel of the condom material and lubricant. Female and male participants reported that satisfaction with stability and sensation during sex and ability to achieve orgasm improved significantly from first to fourth use. At fourth use, female participants reported statistically significant improvement in sensation compared to using nothing. A majority of participants (78%) stated that they would use the Woman's Condom in the future, primarily due to its dual protection profile.* Conclusion*. This study has shown that, in China, the Woman's Condom appears to be acceptable to married couples. User experience contributes to improvement in many aspects of device acceptability.

## 1. Introduction

Female condoms offer dual protection against unintended pregnancy and sexually transmitted infections (STIs) including HIV. Since 2000, several different types of female condoms have become available or are being developed [1, 2]. While short-term acceptability of the FC1 female condom design has been well-documented [3], relatively little data exist on the short-term acceptability of other types of female condoms among various populations.

The Woman's Condom is a second-generation female condom developed by PATH, an international health organization (http://www.path.org/), through a user-centred development process to provide improved acceptability over first-generation products. The Woman's Condom is designed to protect women and couples from unintended pregnancy and STIs including HIV/AIDS and be highly pleasurable and acceptable. In a study involving 60 couples in three countries in 2004, the Woman's Condom design was verified to be comfortable and easy to use, particularly in terms of easy insertion, secure fit during use, good sensation during sex, and easy removal [4]. Further, results from a randomised crossover study among 170 women in Durban, South Africa, that assessed function, safety, acceptability, and preference of the Woman's Condom compared to the FC2 and V-Amour condoms indicated that the three types of female condoms were generally acceptable, and women rated the Woman's Condom better for appearance, ease of use, and overall fit compared to the other two products [5]. In 2008, production technology and product design for the Woman's Condom were transferred to Shanghai Dahua Medical Apparatus Company (Dahua), establishing the foundation for manufacturing and market introduction in China.

This study is the first to assess acceptability of the Woman's Condom in China. Data from this clinical trial were required for product registration for market approval in China. The primary objective of this couples' use study was to assess the acceptability of the Woman's Condom among a sample of couples residing in Shanghai. Data about the performance and safety of the Woman's Condom collected during this study are reported elsewhere [6].

## 2. Materials and Methods

This single-arm couples' use study was conducted in the Shanghai area of China between February and June 2010. The Woman's Condom consists of a very thin, pliable, plastic pouch that easily conforms to the shape of the vagina. It is 22.9 cm (±0.25 cm) long and has a flexible soft outer ring that is designed to hug the external genitalia. Four foam shapes on the outside of the pouch cling lightly to vaginal walls, ensuring stability of the device. The insertion capsule is made from dissolvable polyvinyl alcohol (PVA) and is similar to the PVA used in contraceptive C-Film (Apothecus Pharmaceutical Corporation, New York, NY). The Woman's Condom is packaged dry and not lubricated and comes with a single unit sachet of water-based lubricant. Both the lubricant and the Woman's Condom were manufactured by Dahua (Shanghai, China). The lubricant is packaged in 10.8 cm × 4.4 cm, 3 ml foil sachet with a tapered nozzle at one end.

Prior to initiation of the study, the protocol and informed consent form were approved by the PATH Research Ethics Committee, the Ethics Committee of Shanghai Institute of Planned Parenthood Research, and the Ethics Committee of the Fudan University Hospital of Obstetrics and Gynaecology. All study researchers completed human subjects research training. We recruited women and their partners from four sites affiliated with the Department of Family Planning in the Obstetrics and Gynaecology Hospital of the Medical Centre of Fudan University or the Shanghai Bokang Hospital of Reproductive Medicine. Awareness-raising educational sessions about sexual health and condoms were used as the primary form of recruitment at the two sites. This study was mentioned at the end of the educational session and clients were invited to participate. Interested individuals were given a brief study description and asked to talk to the research team regarding participation. Female participants were enrolled with their male partners to facilitate the collection of data from the male perspective. To be eligible for participation, couples had to be at least 18 years of age, in a monogamous heterosexual relationship with their current partner for the previous six months with the intention of remaining in the same relationship during the study, in good general and genital health, using a nonbarrier method of contraception or not at risk of pregnancy because one partner was sterilized, and without any known sensitivity or allergy to polyurethane or vaginal lubricants. In addition, women could not be pregnant, seeking pregnancy, or breastfeeding.

All female participants were coached in device insertion and removal by a trained researcher. Female participants practised inserting the Woman's Condom at the clinic to ensure that they were comfortable and confident about using the product before being approved to use the product at home with their partner. Couples used the Woman's Condom at home four times over several weeks. After each condom use, women and their male partners completed condom use questionnaires and a coital log and recorded any adverse events in a diary as well as completing an acceptability survey. After the first product use, female participants returned to the clinic and completed a product performance questionnaire. After the fourth product use, both the female and male participants returned to the clinic to complete a second self-administered acceptability survey. At the exit interview, female participants also completed a second product performance questionnaire and evaluated the user instructions (not reported). Both partners participated in a gender-specific exit interview.

The study sample size was based on convenience and conformed to the Shanghai Food and Drug Administration requirement for market registration of the product. Key acceptability endpoints included ease of use, comfort, and satisfaction of the Woman's Condom. Acceptability measures were rated on a five-point Likert scale with response categories ranging from 1 = very easy/comfortable/satisfied to 5 = very difficult/uncomfortable/unsatisfied. Data cleaning and coding and entry and preliminary analysis were conducted in China. Univariate and bivariate analysis of key quantitative variables were conducted using SAS 9.1.3 (Cary, North Carolina). Mean scores were calculated for ordinal acceptability data and a *t*-test was used to compare differences between first and fourth uses in both women and men.

## 3. Results

Sixty couples were enrolled in this study. Ninety-eight percent (*n* = 59) completed all four condom uses between March and April 2010. One couple discontinued early due to scheduling conflicts. The mean age of female and male participants was around forty years (37.8 years and 40 years, resp.). All but two couples were married and living together, and the mean length of their current relationship was 13.3 years. Almost half of the female participants (46%) had completed college/university or above while 32% of the male participants had completed the same level of education. Female participants were employed as company staff (36%), factory worker (29%), government staff (2%), or others (34%). Male participants were employed as factory worker (41%), company staff (36%), government staff (2%), or others (22%). The “others” employment categories include social worker, shopkeeper, and being unemployed. The current contraceptive method used by most participants was intrauterine devices (76%), followed by oral contraceptives (24%). Few (12%) participants reported current use of dual methods (IUD plus the male condom). Ever use of male condoms was slightly over half for female participants and almost 60% for male participants. Less than five percent of either female or male participants reported ever use of spermicide or female condoms.

### 3.1. Acceptability

The two aspects of the Woman's Condom that women most commonly reported as liking most were stability during sex and ease of removal. The two aspects of the Woman's Condom that women most commonly reported as liking least were insertion and appearance of the device on their body. Women reported the pouch material and lubricant as being the two device features that they liked the most. They reported the outer ring as being the device feature that they liked the least.

### 3.2. Female Use Behavior

Female participants reported that the Woman's Condom was generally easy to use. A majority of female participants reported that handling the condom (68%), following instructions (67%), and removing the condom (61%) were very easy. Ease of insertion and the convenience of lubrication were considered less easy aspects of Woman's Condom use. The majority of women (83%) reported inserting the Woman's Condom 1–5 minutes before sex by lying on their backs. The male partner assisted the women with insertion in nine instances during first use and thirteen instances during fourth use. At first use, the majority of female participants (64%) reported no problems with insertion. Eleven women reported that they were unsure how deeply to insert the device and if the device was correctly placed, six women reported difficulty inserting the device in their vagina, and four women reported discomfort during insertion.

Women reported wearing the device less than fifteen minutes (54%), between 15 and 30 minutes (42%), or more than thirty minutes (4%). Most women (83%) used the twist-and-pull method to remove the device. All women disposed of the Woman's Condom in the trash and only one woman stated that she was concerned about disposing of the condom in this way.

### 3.3. Ease of Use

All acceptability measures (ease of use, comfort, and satisfaction) improved for both female and male participants from first to fourth use (Table 1). Male participants reported that ease of handling, inserting, and removing the device improved significantly from first to fourth use.

### 3.4. Comfort

Both female and male participants reported that comfort during insertion, feel of lubricant during insertion, comfort/fit of ring during use, and overall comfort during use improved significantly from first to fourth use. In addition, at fourth use, female participants reported statistically significant improvement in the feel of the condom material and the feel of the lubricant during use.

### 3.5. Satisfaction

Both female and male participants reported that satisfaction with stability during sex, satisfaction with sensation during sex, and ability to achieve orgasm improved significantly from first to fourth use. Female participants reported statistically significant improvement in the sensation/stimulation compared to nothing from first to fourth use.

### 3.6. Lubricant Use Behavior

Since the Woman's Condom is not prelubricated, the instructions for use recommend applying lubricant to the inside of the pouch after it is inserted in the vagina and before penile insertion. At first use, 86% of women reported that they had applied lubricant to the Woman's Condom before sex. Of these women, 96% stated that they used only “some” of the lubricant in the sachet. Various methods were used to apply the lubricant including putting the lubricant on the finger, applying the lubricant directly from the package onto the condom pouch and then spreading it with the finger, putting the lubricant on the partner's penis, and applying the lubricant directly from the package into the condom pouch. Six percent of the women reported that they applied more lubricant to the condom after sex started. Most women (59%) stated that the lubricant had no effect on the quality of their sexual experience while 19% reported that use of the lubricant enhanced their sexual experience. Only one woman stated that lubricant use detracted from her sexual experience. At fourth use, 68% of women reported that the lubricant enhanced the ease of having sex.

### 3.7. Product Preference

At the study exit interview, participants were asked a range of questions related to product preference and future use (Table 2). While women and men did not report a clear preference for a female over a male condom, a majority (78%) stated that they would use the Woman's Condom in the future, primarily due to its dual protection profile. Women cited the benefits of using a female condom as being contraceptive protection (95%), STI protection (93%), female empowerment (34%), lack of side effects (31%), an option when male condoms are refused (22%), ability to insert it in advance of sex (19%), and an increase in sexual pleasure (10%). Men reported the benefits of using a female condom as being contraceptive protection (80%), STI protection (78%), an option when male condoms are refused (20%), female empowerment (20%), lack of side effects (17%), ability to insert it in advance of sex (12%), and an increase in sexual pleasure (5%). A similar number of female and 69% of male participants would recommend use of the Woman's Condom to a friend. Most female (88%) and male (80%) participants also stated that they would purchase the Woman's Condom if the price was affordable to them.

## 4. Discussion

This is the first study to assess acceptability of the Woman's Condom in China. To date, relatively few studies in China have assessed female condom acceptability, and those studies have focused on sex workers. Acceptability of other types of female condoms has been demonstrated by women in sex establishments in rural and small urban areas of southern China [7, 8], sex workers in Enping City, China [9], and female STI patients attending a government STI clinic in Hong Kong [10]. Data from the current study suggest good overall acceptability of the Woman's Condom in a general population of married couples recruited from family planning clinics. These results suggest a strong market opportunity for the Woman's Condom, given that married couples in China are a very different population segment compared to female sex workers.

In this study, acceptability measures improved for both women and men with increased user experience. This shift in acceptability from first to fourth use is consistent with the female condom learning curve noted in other Woman's Condom studies [4] and studies of other female condoms as well [11, 12]. Evidence of the learning curve phenomenon for acceptability measures has not been documented rigorously. For example, almost all (93.5%) of the 155 Chinese sex workers enrolled in the intervention arm of a study expressed that their sexual satisfaction had increased with their familiarity with the female condom [13].

Most women reported applying lubricant inside of the pouch before sex per the instructions, and women reported that the lubricant made sex easier. This was an unexpected surprise since formative research and market research undertaken to prepare for this study had previously suggested that personal lubricants are not widely used by couples in China, may not be culturally acceptable, and are used only by older women and that women would be unwilling to apply lubricant using their fingers. In contrast, women in this study used a variety of strategies to apply lubricant and reported lubricant as one of the two features they liked the most about the Woman's Condom.

Female condoms are a relatively unknown product in China. None of the couples recruited for this study had seen or heard of female condoms before, and only slightly more than half had experience using male condoms. However, this population of married couples showed interest in using the Woman's Condom and recommending it to others, especially for dual protection. Male partner involvement is often the key to successful female condom use. This population of married women in China may be able to benefit from the preference of their male partner for this dual protection method. Behavior change communication messaging related to dual protection with a clear focus on STI prevention and product safety could be instrumental in promoting use of this product.

The results from this study are limited in that these couples were primarily married, were living together, had relationships of long duration, and ranged in age from 25 to 57 years. In view of these particular circumstances, our findings may not necessarily apply to populations with different characteristics.

## 5. Conclusion

This study has shown that, in China, the Woman's Condom appears to be acceptable to married couples, especially couples who are interested in dual protection. Improvement in acceptability parameters from first to fourth use indicates that device familiarity enhances user perception as well as a manageable learning curve. User experience contributes to improvement in many aspects of device acceptability.

## Figures and Tables

**Table 1:** Acceptability of Woman's Condom by female and male participants at first and fourth uses.

Acceptability measure	Women (*n* = 59)			Men (*n* = 59)		
	First-use mean (SD)	Fourth-use mean (SD)	*p* value	First-use mean (SD)	Fourth-use mean (SD)	*p* value
*Ease of use* *1 = very easy* *5 = very difficult*						
Ease of following instructions	1.32 (0.90)	1.25 (0.54)	0.6094	1.24 (0.88)	1.27 (0.67)	0.8353
Ease of handling condom	1.36 (0.66)	1.24 (0.50)	0.2679	1.42 (0.81)	1.00 (0.69)	0.0030^*∗*^
Ease of inserting condom	1.95 (1.04)	1.76 (0.92)	0.2954	1.86 (0.99)	1.29 (1.07)	0.0033^*∗*^
Convenience of lubrication	1.53 (0.80)	1.44 (0.77)	0.5348	1.54 (0.82)	1.34 (0.82)	0.1879
Ease of removing condom	1.32 (0.60)	1.31 (0.53)	0.9237	1.36 (0.66)	1.07 (0.74)	0.0266^*∗*^
Ease of use (general)	1.85 (0.98)	1.54 (0.70)	0.0504	1.81 (0.96)	1.53 (0.80)	0.0879

*Comfort* *1 = very comfortable* *5 = very uncomfortable*						
Comfort during insertion of condom or penis into condom	2.32 (0.84)	1.81 (0.70)	0.0005^*∗∗*^	2.31 (1.00)	1.86 (0.73)	0.0061^*∗∗*^
Feel of lubricant during insertion of condom or penis into condom	2.11 (0.98)	1.76 (0.75)	0.0314^*∗*^	2.14 (1.00)	1.73 (0.76)	0.0135
Feel of condom material	2.17 (1.00)	1.81 (0.96)	0.0484^*∗*^	2.12 (0.97)	1.81 (0.84)	0.0660
Overall comfort during use	2.29 (1.00)	1.85 (0.81)	0.0098^*∗∗*^	2.15 (0.91)	1.84 (0.67)	0.0373
Comfort/fit of ring during use	2.34 (1.08)	1.98 (0.81)	0.0428^*∗*^	2.36 (1.06)	1.97 (0.76)	0.0234
Feel of lubricant during use	2.16 (1.00)	1.75 (0.71)	0.0115^*∗*^	2.10 (0.95)	1.80 (0.83)	0.0703
Comfort during removal of condom or penis from condom	2.05 (1.02)	1.73 (0.72)	0.0514	2.12 (1.07)	1.86 (0.80)	0.1377

*Satisfaction* *1 = very satisfactory* *5 = very unsatisfactory*						
Satisfaction with stability during sex	1.97 (0.85)	1.54 (0.75)	0.0043^*∗∗*^	1.92 (0.97)	1.47 (0.60)	0.0030^*∗∗*^
Satisfaction with sensation of sex	2.44 (0.98)	1.75 (0.71)	<0.0001^*∗∗*^	2.25 (0.99)	1.88 (0.79)	0.0267
Ability to achieve orgasm	2.47 (1.04)	1.93 (0.83)	0.0023^*∗∗*^	2.29 (1.03)	1.86 (0.75)	0.0108
Sexual satisfaction compared to male condom	1.80 (1.17)	1.58 (0.93)	0.2605	1.78 (1.18)	1.69 (0.88)	0.6395
General use compared to male condom	1.88 (1.13)	1.64 (0.89)	0.2025	1.90 (1.16)	1.73 (0.85)	0.3658
Sensation/stimulation compared to nothing	2.51 (0.97)	2.03 (0.79)	0.0039^*∗∗*^	2.56 (1.04)	2.44 (1.07)	0.5380

^*∗*^Significant at the *p* < 0.05 level.

^*∗∗*^Significant at the *p* < 0.01 level.

**Table 2:** Product preference and willingness to use by sex (number [%]).

	Women *n* = 59	Men *n* = 59
*How does using this device compare to using a male condom? *		
Much better	34	24
Better	31	31
Same	17	32
Worse	6	6
Never used a male condom before	14	8

*Would you prefer to use a male or female condom?*		
Male condom	10	12
Female condom	31	32
No preference	36	41
Neither	8	7
Never used a male condom	15	8

*Would you use this female condom in the future?*		
No	22	22
Yes	78	78

*Why? (n = 49)*		
STI/HIV prevention	9	20
Contraception	2	4
Both	85	72
Other	4	4

*Would you recommend use of this female condom to a friend?*		
No	22	31
Yes	78	69

*What type of information do you think someone who has never used this female condom would need before they would feel comfortable using it? (multiple responses allowed)*		
Pregnancy prevention information	59	56
STI prevention information	68	69
Safety information	75	44
Articles or ads in magazines or newspapers	17	19
Other	0	2

*If you needed to purchase a condom, would you purchase this female condom if the price were affordable to you?*		
No	12	20
Yes	88	80

*If you were going to purchase/use this female condom, where would you want to buy it?*		
Commercial outlets	34	39
Hospital outlets	59	54
Family planning station in community	61	53
Internet	3	12
Other	0	0
